# Quantitative assessment of relationship between sequence similarity and function similarity

**DOI:** 10.1186/1471-2164-8-222

**Published:** 2007-07-09

**Authors:** Trupti Joshi, Dong Xu

**Affiliations:** 1 Digital Biology Laboratory, Department of Computer Science and Christopher S. Bond Life Sciences Center, University of Missouri, Columbia, Missouri 65211, USA

## Abstract

**Background:**

Comparative sequence analysis is considered as the first step towards annotating new proteins in genome annotation. However, sequence comparison may lead to creation and propagation of function assignment errors. Thus, it is important to perform a thorough analysis for the quality of sequence-based function assignment using large-scale data in a systematic way.

**Results:**

We present an analysis of the relationship between sequence similarity and function similarity for the proteins in four model organisms, i.e., *Arabidopsis thaliana, Saccharomyces cerevisiae, Caenorrhabditis elegans*, and *Drosophila melanogaster*. Using a measure of functional similarity based on the three categories of Gene Ontology (GO) classifications (biological process, molecular function, and cellular component), we quantified the correlation between functional similarity and sequence similarity measured by sequence identity or statistical significance of the alignment and compared such a correlation against randomly chosen protein pairs.

**Conclusion:**

Various sequence-function relationships were identified from BLAST versus PSI-BLAST, sequence identity versus Expectation Value, GO indices versus semantic similarity approaches, and within genome versus between genome comparisons, for the three GO categories. Our study provides a benchmark to estimate the confidence in assignment of functions purely based on sequence similarity.

## 1. Background

Large-scale genome sequencing projects have discovered many new proteins. Of all the proteins whose sequences are known, functions have been experimentally determined for only a small percentage [1]. Annotation of a genome involves assignment of functions to proteins in most cases on the basis of sequence similarity. Protein function assignments based on postulated homology as recognized by sequence identity or significant expectation value of alignment are used routinely in genome analysis. Over the past years, many computational methods [2-11] have been developed to predict function through identifying sequence similarity between a protein of unknown function and one or more proteins with experimentally characterized or computationally predicted functions. However, it is widely recognized that functional annotations should be transferred with caution, as the sequence similarity does not guarantee evolutionary or functional relationship. In addition, if a protein is assigned an incorrect function in a database, the error could carry over to other proteins for which functions are inferred by sequence relationship to the protein with errant function assignment [12-14].

**Figure 1:**
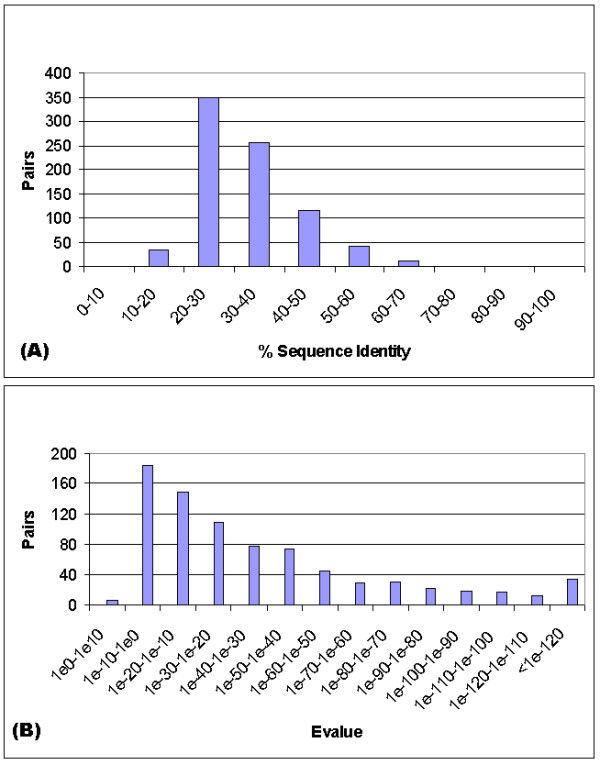
Distribution of yeast and *Arabidopsis *unique orthologous pairs from COGs against sequence identity and expectation value intervals.

Despite the central role that sequence comparison programs play in functional annotation, a thorough analysis of the quality of methods based on a large-scale dataset has not been performed. Improvements in the sensitivity of sequence comparison algorithms have reached the point that proteins with previously undetectable sequence relationship, for instance with 10–15% identical residues, may be classified as similar [15]. On the other hand, alignments are more likely to be correct for higher levels of pairwise sequence identity; and are less likely to be correct in the so-called "twilight zone", where the sequence similarity is low [16]. An estimate of the expectation value of an alignment provides a good assessment for whether the two aligned proteins are homologous [17]. Nevertheless, prediction of protein function from sequence is a difficult problem, because not only sequence similarity does not guarantee homology, but also homologous proteins often have different functions [18,19]. In particular, when two proteins are distantly related, there is no good indicator to reliably assess whether they are homologous or not. Figure 1 shows the number of unique orthologous pairs between the yeast *Saccharomyces cerevisiae *and *Arabidopsis thaliana *acquired from the Website of Clusters of Orthologous Groups of proteins (COGs) [37]. The COG pairs distribute in a broad range of sequence identity and expectation value. It is clear that neither percentage of sequence identity nor expectation value can give a complete insight into the relationship between the two proteins. Towards this we wish to study the detailed quantitative relationship in terms of functions and relate it with sequence identity and expectation value intervals.

A number of studies in sequence-function relationship have been carried out. Shah et al. [20] showed that many EC (Enzyme Commission) classes could not be perfectly discriminated by sequence similarity at any threshold. Pawlowski et al. [15] have studied the relation between sequence similarity and functional similarities based on the EC classification for the *E. coli *genome. However, this study is limited only to within genome comparisons and lacks any analysis based on inter-genome comparisons. Devos et al. [21] have studied the complexity in transferring function between similar sequences. Their study shows that binding site, keywords, and functional class annotations are less conserved than EC numbers, and all of them in turn are less conserved than protein structure. Wilson et al. showed that percent identity in sequence alignment is more effective at quantifying functional conservation of their simple classification of SCOP domains than modern probabilistic scores [22]. However, all these studies did not use a broad definition of functions for a systematic large-scale analysis. In this paper, we will build a comprehensive and systematic benchmark for the sequence-function relationship using four model organisms (*Arabidopsis thaliana, Saccharomyces cerevisiae, Caenorrhabditis elegans*, and *Drosophila melanogaster) *and controlled vocabularies of function annotation terms in the Gene Ontology [38] from three different perspectives, i.e., biological process, molecular function, and cellular component.

## 2. Results and discussion

**Figure 2:**
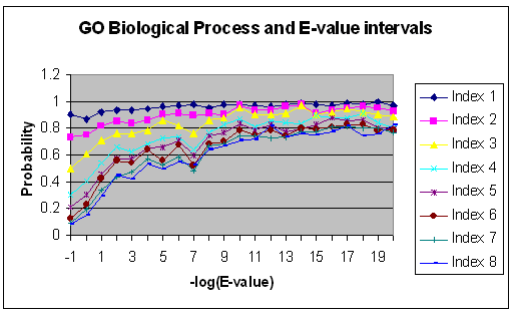
Relation between functional similarity in terms of the GO Indices and the negative logarithmic (base 10) E-value of sequence similarity within the same genomes using FASTA for the GO Biological Process Annotations.

**Figure 9:**
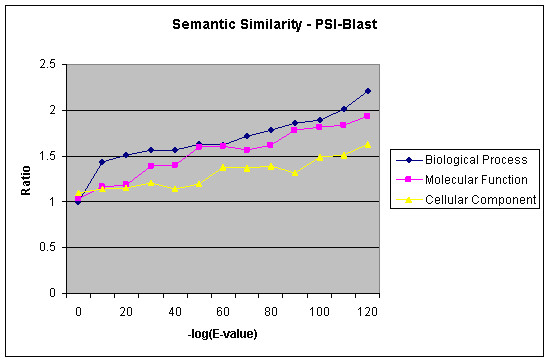
Relation between functional similarity for GO Biological Process, Molecular Function and Cellular Component Annotations vs. E-value intervals (negative logarithmic with base 10) within the same genomes using PSI-BLAST.

The sequence comparisons within and across the four genomes provide a global view on the relationship between sequence similarity and function similarity. Figure 2 shows a consistent correlation between function similarity of biological process at different GO index levels and the Expectation Values (E-values) of sequence alignment using BLAST [23]. There is also a higher functional similarity for the lower GO Index levels in comparison to the higher Index levels. In particular, at levels 1 and 2, the function similarity reaches very high even when the sequence similarity is insignificant. This is mainly due to the fact that many more genes can be found under a GO Index of lower level than of higher level, and hence, there is a higher chance for two randomly picked genes to share the GO Index at the lower level (see Figure 9 and related discussion).

**Figure 3:**
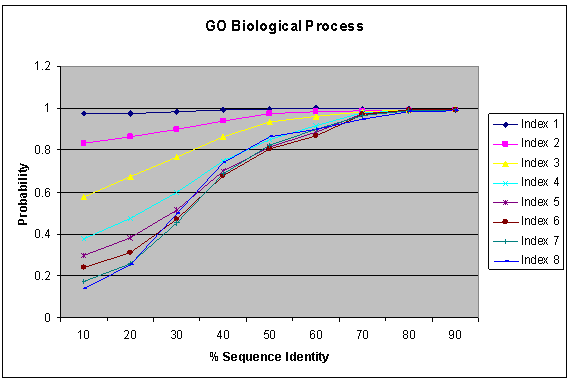
Relation between percentage of sequence similarity and functional similarity for GO Biological Process Annotations within the same genomes using BLAST.

**Figure 4:**
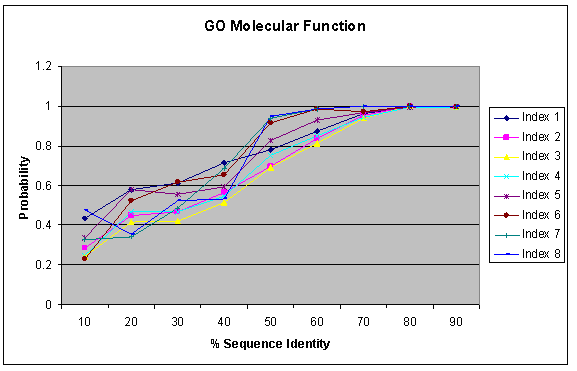
Relation between percentage of sequence similarity and functional similarity for GO Molecular Function Annotations within the same genomes using BLAST.

**Figure 5:**
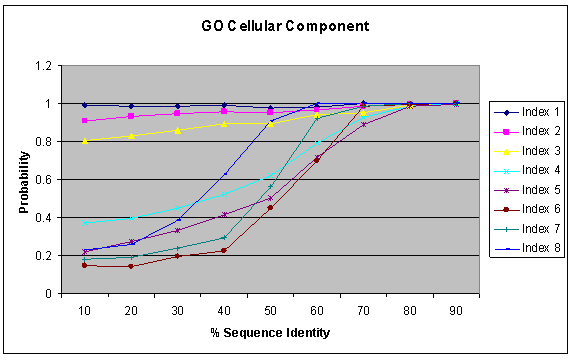
Relation between percentage of sequence similarity and functional similarity for GO Cellular Component Annotations within the same genomes using BLAST.

Figure 3 shows the result of function similarity with respect to sequence identity as identified by the BLAST for GO Biological Process annotations. It shows that probability of functional conservation increases with increasing sequence conservation. A similar trend is observed in different GO Index levels as in Figure 2. The probability is based on the number of pairs sharing the same function at a certain index level against the total pairs having any functions at the respective index level for a given sequence similarity interval. Such per index probability may sometimes result in higher probability for higher index levels (probably due to limited sample size) and lead to the cross-over between curves from various index levels. Interestingly, high sequence identity is a better indicator of function similarity than significant E-value as used in Figure 2. If two proteins have sequence identity more than 70%, they have about 90% probability or more to share the same biological process for GO index levels 1–8. On the other hand, E-value depends on many factors, in particular the lengths of the two proteins. For large proteins with homologous relationship, the E-value tends to be more significant for computational identification of the homology relationship, but their sequence identity can be very weak and their functional relationship may be remote. Figures 4 and 5 show similar results as Figure 3 for GO Molecular Function and GO Cellular Component Annotations, respectively. The result is similar to that observed by Pawlowski et al. in their studies on enzymes based on the *E. coli *genome [15] and by Wilson et al [22] who use FLY+ENZYME classification SCOP domains, MIPS and GenProtEC to study sequence and functional conservation.

**Figure 6:**
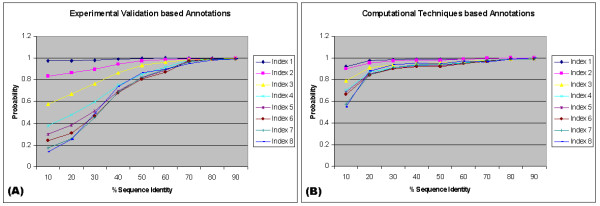
Functional conservation patterns for GO Biological Process annotations (A) based on evidences from experimental validations and (B) based on computational techniques such as electronic annotations, against percentage of sequence similarity.

Functional conservation measures from GO annotations based on computational techniques such as electronic annotation based on sequence similarity has a behavioral pattern completely different from Figures 3, 4 and 5. When GO Biological process annotations are made based on evidences from experimental validations (Figure 6A), such proteins tend to conserve and share functions with higher probability for pairs with high sequence identity as compared to pairs with remote sequence similarity. In all cases, when a pair of proteins share sequence identity 30% or less, the chance for them to share any of the three GO categories at high levels is about 50% or less. However, this pattern is lost when annotations are made purely based on computational techniques (Figure 6B) and the functions are conserved with almost equal probabilities irrespective of the sequence conservation. This depicts the difference in the quality of these two annotations, and indicates that many annotations based on computational techniques may be incorrect. Some of these incorrect annotations could be due to over-extension of functional details when inferring a query protein from a protein hit with known function. For example, a protein in two-component signal transduction system (GO:0000160) could be predicted to cell surface receptor linked signal transduction (GO:0007166), although both proteins are in signal transduction (GO:0007165). The trend stands true for both Molecular Function and Cellular Component (data not shown).

**Figure 7:**
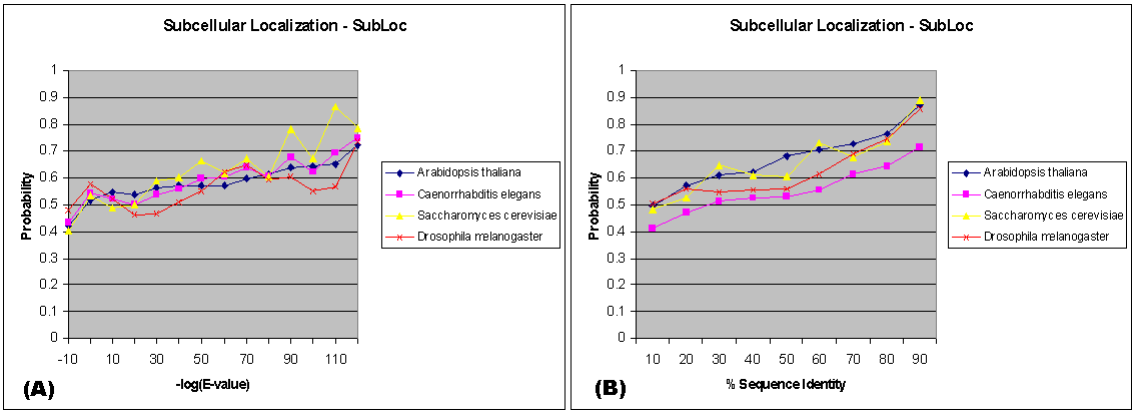
**A**. Relation between E-value intervals (negative logarithmic with base 10) of seq uence similarity and similarity in SubLoc predicted localization of proteins within the same genomes using FASTA. **B**. Relation between percentage of sequence similarity and similarity in SubLoc predicted localization of proteins within the same genomes using BLAST.

We also compared the SubLoc predicted localizations for all the proteins across genomes. Figure 7 shows the localization similarity *versus *the sequence similarity in terms of E-value and percentage of sequence identity for intra-genome comparisons within four genomes. In this case the localization is measured by five types as described in Section 4.4, instead of the GO Cellular Component Annotation, a detailed level that no existing software can predict reliably. Subcellular localization conservation shows similar results when compared in terms of E-value or sequence identity. Inter-genome comparisons based on the predicted subcellular localizations also behave in a manner similar to the intra-genome comparisons (data not shown). It is interesting to note that the behavior of the curves of the four genomes is similar in respect of E-value (Figure 7A). On the other hand, the behavior of the curves of the four genomes shows the difference in respect to the sequence identity (Figure 7B), in particular, *Caenorrhabditis elegans *shows significantly more divergence in localization under the same sequence identity than the other three genomes.

**Figure 8:**
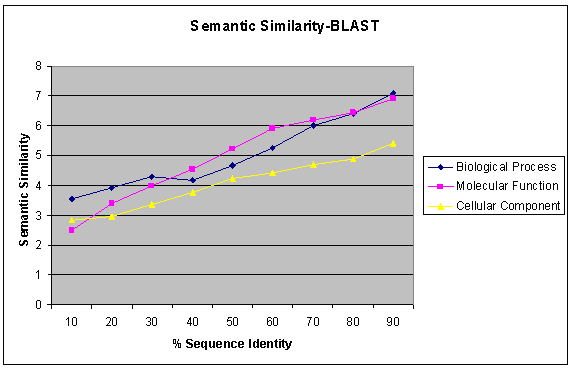
Relation between Semantic similarity and sequence identity for GO Annotations for combined inter and intra genome comparisons using BLAST.

We also calculated functional similarity in terms of semantic similarity between the functional annotation terms of GO (see section 4.3) using BLAST. Figure 8 shows the relationship between semantic similarity and sequence identity for all four genomes combined for all three Ontologies. The semantic similarity measures remove the bias seen between different levels of indices of the ontology. Figure 9 shows the relationship between remote homologs using PSI-BLAST [24] and semantic similarity. For many of the PSI-BLAST pairs, the sequence identity is below 30%. Hence, we focus the sequence-function relationship based on E-value, instead of sequence identity.

**Figure 10:**
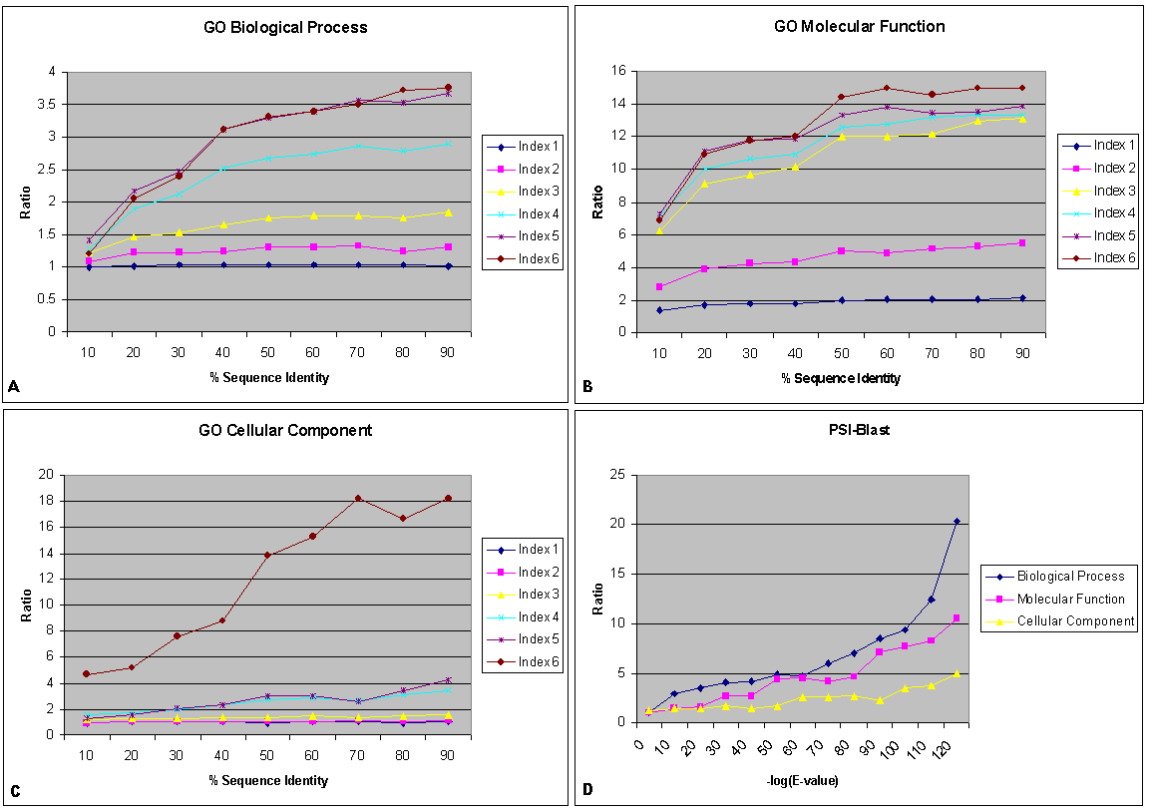
Relation between percentage of sequence similarity and functional similarity for GO (A) Biological Process, (B) Molecular Function and (C) Cellular Component Annotations within the same genomes using BLAST and (D) for all annotations using PSI-BLAST respectively, in the form of normalized ratio of pms(t1, t2), which is the probability of the minimum subsumer for terms t1 and t2 (see section 4.3).

We have also computed results as described above for any random pairs with known function annotation. Then, we calculated a normalized ratio of function similarity in terms of sequence identity by comparing the results in Figures 3 through 5 against similar results from random pairs. Figure 10 shows the normalized ratio results for GO Biological Process, Molecular Function and Cellular Component Annotations in subplots A, B and C, respectively. Our results clearly show that the normalized ratio increases for higher sequence identity intervals as well as higher levels of shared GO Indices, highlighting the higher chance of functional conservation over randomly chosen pairs for these groups. GO annotations for Index level 3 and above are very informative as the probability of correct functional assignment based on sequence similarity is significantly above random. Figure 10D shows normalized results for all three annotations using PSI-BLAST in subplot D. It indicates that PSI-BLAST has substantial enrichment of function assignment for function prediction. This may be because PSI-BLAST utilizes multiple sequence profiles that enhance the recognition of the sequence-function relationship.

**Figure 11:**
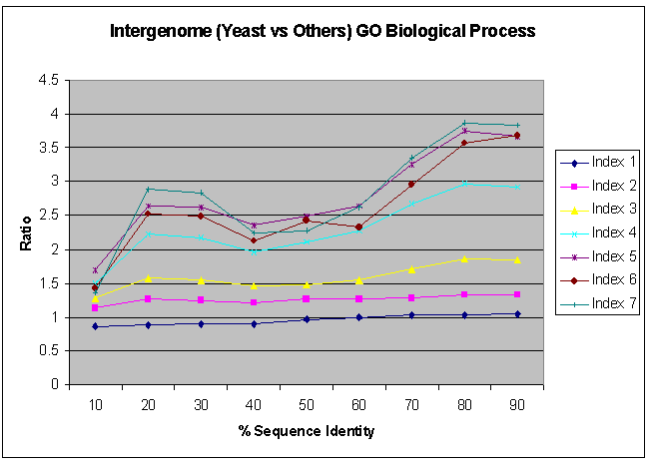
Relation between percentage of sequence similarity and functional similarity for the GO Biological Process Annotations for inter-genome comparison of yeast ORFs against others using BLAST, in the form of normalized ratio.

**Figure 12:**
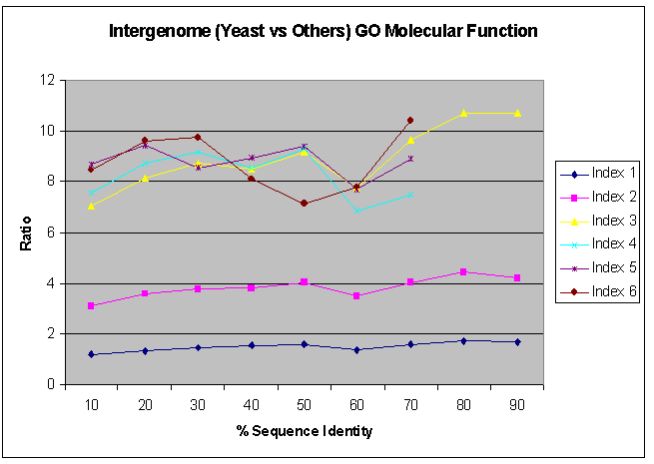
Relation between percentage of sequence similarity and functional similarity for the GO Molecular Function Annotations for inter-genome comparison of yeast ORFs against others using BLAST, in the form of normalized ratio. Data points with a sample size less than 10 gene pairs are not sure, as the statistics is not significant.

Figure 11 and 12 show results similar to Figure 10 for normalized ratio against random pairs for inter-genome sequence similarity comparisons between each yeast protein and the other three genomes, for GO Biological Process and Molecular Function Annotations, respectively. It appears that the trend of biological process in inter-genome comparison is similar to the one in intra-genome comparison, while the trend of molecular function in inter-genome comparison is very diverse. This suggests that many homologous genes may have evolved into different molecular functions in different genomes.

## 3. Conclusion

It has been long recognized that genome annotations using computational methods produce many false function assignments. Many of these methods have been applied to function prediction. They often provide valuable hypotheses, but none are perfect. As a result, it is known that many databases contain incorrect function assignments, and these erroneous assignments propagate from one database to another. Nevertheless, up until now there has been no systematic study for this critical issue. The question whether two proteins are functionally similar is very complex to answer. Function is a very complex notion involving many different aspects including chemical, biochemical, cellular, organism mediated, and developmental processes. Qualitatively it is expected that with higher sequence similarity, the two proteins are more likely to have related functions. However, quantitatively the relationship between function similarity at the different categories and sequence similarity has not been studied deeply. Such a quantitative study is fundamentally important, as it can provide assessment of gene function prediction quality and insights into the underlying mechanisms of new evolving functions through changes in sequence [25,26].

Our study confirms that sequence comparison often provides good suggestions for gene functions or related functions. These suggestions serve as useful hypotheses for further experimental work to confirm, refine or refute the predictions. Such a process can substantially increase the speed of biological knowledge discovery. On the other hand, when assigning function based purely on similarity to proteins of known function (as annotated in databases), it is important to be aware of incomplete or wrong annotations. Given the value of computational function annotation, our study also shows that a significant portion of gene annotations of biological process, molecular function, and cellular component based solely on sequence similarity, in particular, when the sequence similarity is low, are unreliable. Our study also provides a numerical benchmark for the extent to which one can trust computational annotation. It is possible that a confidence score can be derived from our study for any annotation based on sequence similarity. With this score in the annotation file, the user can have a better insight about the quality of the annotations. Furthermore, our analyses highlights the different sequence-function relationships identified from BLAST versus PSI-BLAST, sequence identity versus Expectation value, GO indices versus semantic similarity approaches and within genome versus between genome comparisons, for the three GO classification types.

There are some limitations in our current study. Our study can only reflect certain aspect of protein function. Protein function variations may result from factors other than sequence, such as alternative splicing and post-translational modification, and our method does not address these factors. Another limitation is that when we assess gene function prediction, we only consider one hit at a time in a database. In many cases, sequence comparison yields multiple hits for one query protein and these hits may have different functions. In our future study, we will develop a new method to assess the function prediction for a query protein by combining the functions of multiple hits while considering the dependence among these functions and the E-values of the hits.

## 4. Methods

### 4.1 Protein sequence databases

We selected the genomes of *Arabidopsis thaliana, Saccharomyces cerevisiae, Caenorrhabditis elegans *and *Drosophila melanogaster *for the study. All four genomes are well-studied model organisms in eukaryotes. The complete set of *Arabidopsis thaliana *protein sequences for 27,288 ORFs was acquired from The Arabidopsis Information Resource (TAIR) [39]. We also obtained proteins sequences for 21,588 *Caenorrhabditis elegans *ORFs, 6350 *Saccharomyces cerevisiae *ORFs and 13,665 *Drosophila melanogaster *ORFs from NCBI [40]. Table 1 lists the number of ORFs for all the four genomes whose functions are annotated based on experimental evidences or sequence similarity measures for all the three functional categories.

**Table 1:** Details about the four genomes and number of functional annotations in biological process, molecular function and cellular component assigned based on experimental or sequence similarity evidence

		**# of annotations verified by experimental evidence **			**# of annotations based on computational methods**		
		
**Species**	**# of ORFs**	**Biological**	**Molecular**	**Cellular**	**Biological**	**Molecular**	**Cellular**
*Arabidopsis thaliana*	27,288	2245	817	751	9602	13,903	15,031
*Caenorrhabditis elegans*	21,588	826	112	265	3691	5149	2597
*Saccharomyces cerevisiae*	6350	3885	3003	4554	2230	3331	1445
*Drosophila melanogaster*	13,665	1361	781	677	2840	4102	2653

### 4.2 Protein functional classification

The Gene Ontology (GO) functional classification [27] has three functional categories, i.e., biological process, molecular function and cellular component. It is not a hierarchical tree but the directed acyclic nature of the graph can be well captured in a series of numerical numbers. We have generated a numerical GO INDEX for all three classifications individually, which represents the structure of every ontology. The deepest level of index is 13. A GO Index, as denoted by numbers, e.g. 1-4-2-29, characterizes the function of every protein. The first number corresponds to the type of functional category, e.g. 1 represents biological process, 2 represents molecular function and 3 represents cellular component. The subsequent numbers correspond to subcategories describing the type of function or localization in increasing detail. The higher the GO Index level, the more specific is the functional category the protein belongs to. Table 2 shows an example of GO indices.

**Table 2:** Example of GO index and the corresponding GO ID and functional category

**Index Level**	**GO Index**	**Functional category and GO ID**
Index 1	1–2	cellular process (GO:0009987)
Index 2	1-2-1	cell communication (GO:0007154)
Index 3	1-2-1-8	signal transduction (GO:0007165)
Index 4	1-2-1-8-1	cell surface receptor linked signal transduction (GO:0007166)
Index 5	1-2-1-8-1- 4	G-protein coupled receptor protein signaling pathway (GO:0030454)

We assume that the functional relationship between two proteins is reflected by the number of index levels that they share. We have demonstrated the usefulness of such an assumption in our early studies for gene function prediction [28,29]. We acquired the GO annotations for all the genes in the four genomes and for the three functional categories from GO Website [38]. A gene can (and usually does) belong to multiple indices at various levels in the graph, as proteins may be involved in multiple functions in a cell. Different indices could correspond to the same GO term as well.

Gene Ontology annotation is based on various evidences to annotate functional categories. Towards quality control, all the plots (except for Figure 6B) presented in this paper are based on the annotations with actual experimental evidences such as IDA (inferred from direct assay), IEP (inferred from expression pattern), IGI (inferred from genetic interaction), IMP (inferred from mutant phenotype), IPI (inferred from physical interaction), RCA (inferred from reviewed computational analysis) and TAS (traceable author statement). We performed some comparisons using annotations assigned purely based on computational methods such as ISS (inferred from sequence similarity) and IEA (inferred from electronic annotation), but the plots are not presented here. We have removed the functional annotations that were purely based on evidences such as ND (no biological data available) and NAS (non-traceable author statement.

### 4.3 Protein functional similarity

Within each family of proteins with similar sequences, functional similarity between proteins is expressed as the number of common roots shared by their functional classification other than the first level, which represents a classification of biological process, molecular function and cellular component. In the case of proteins with multiple functional assignments, the maximum indices of overlap are considered. For example, consider a gene pair ORF1 and ORF2, both annotated proteins. Assume ORF1 has a function represented by GO INDEX 1-1-3-3-4 and ORF2 has a function 1-1-3-2. When compared with each other for the level of matching GO INDEX, they match through INDEX level 1 (1-1) and level 2 (1-1-3) and will have functional similarity equal to 2. The functional similarity defined this way can assume values from 1 to 12.

**Figure 13:**
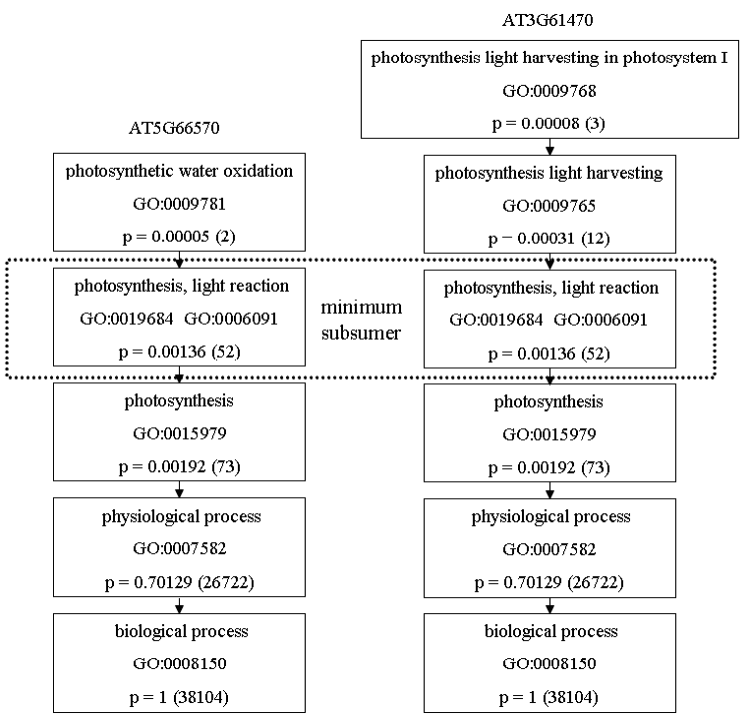
GO Biological Process sub-graph with probabilities and minimum subsumer. The numbers in parentheses denote the occurrence of the GO term and any of its descendants in the GO.

We also calculate functional similarity in terms of semantic similarity between the GO functional annotation terms [30,31]. An example of calculating the probabilities is shown in Figure 13. To calculate semantic similarity between the protein pairs, the probability of each term assigned to the gene product is first derived. For each gene in the organism, the probability is calculated by counting the number of the descendants of an assigned GO term plus 1 (the GO term itself), divided by the total number of GO term annotations in the organism. The probability of each node increases as we go towards the root of the GO ontology, which is defined as "Biological Process" (GO:0008150), "Molecular Function" (GO:0003674) or "Cellular Component" (GO:0005575) in the three Ontologies and has a probability of 1. The semantic similarity between ontology terms is defined as:

*SS *(*t*_1_,*t*_2_) = -ln *p*_*ms*_(*t*_1_,*t*_2_)

where, *p*_*ms*_(t_1_, t_2_) is the probability of the minimum subsumer for terms t_1 _and t_2_. The minimum subsumer for terms t_1 _and t_2 _is defined as the common parent of the deepest GO Index level shared by t_1 _and t_2_.

### 4.4 Protein subcellular localization

The subcellular distribution of proteins within a proteome is useful and important to a global understanding of the molecular mechanisms of a cell. Protein localization can be seen as an indicator of its function. Localization data can be used as a means of evaluating protein information inferred from other resources. Furthermore, the subcellular localization of a protein often reveals its activity mechanism. The subcellular localization information was predicted using SubLoc [32,33,41]. The five main subcellular localization categories as predicted by SubLoc are Cytoplasmic, Nuclear, Mitochondrial, Transmembrane, and Extracellular. The total numbers of proteins with predicted subcellular localization are 6323 in *Saccharomyces cerevisiae*, 27,288 in *Arabidopsis thaliana*, 21,588 in *Caenorrhabditis elegans*, and 18,498 in *Drosophila melanogaster*. It is worth mentioning that the subcellular localization predictions were not based on sequence similarity.

### 4.5 Protein sequence similarity search

The sequence similarity search was done using tools such as BLAST [23], FASTA [34,35] and PSI-BLAST [24]. BLAST is the most widely used sequence comparison tool, particularly for genome annotation. FASTA is more sensitive in accuracy but slower than BLAST. Both FASTA and BLAST were developed for pairwise local alignment, with heuristics used. PSI-BLAST is used to identify remote homology based on iterative BLAST searches.

We compared the sequences for within as well as between genome sequence similarities. Each protein sequence was compared against the complete set of proteins for the same genome for within genome comparisons. For between genome comparisons, a pair of similar protein pair was identified using the reciprocal search method [36], i.e., the two proteins in the pair are the best hits in each other's genome from sequence search. Intra-genome sequence comparison would reflect the sequence similarity between the paralogs; while the inter-genome comparison would partially highlight the orthologous sequence similarities.

To assess the significance of a sequence comparison, an expectation value or E-value can be calculated. This value represents the number of different alignments with the observed alignment score or better that are expected to occur in the database search simply by chance. The E-value is a widely accepted measure for assessing potential biological relationship, as it is an indicator of the probability for finding the match by chance. Smaller E-values represent more likelihood of having an underlying biological relationship. In this study, we will use both E-value and sequence identity as parameters to quantify sequence similarity. On the other hand, E-values depend on a number of computational factors, such as the length of the query protein and the size of search database. The issues prevent the E-value from being a reliable indicator for homology, as addressed in Fig. 1 and related discussions.

### 4.6 Availability

The data and results are publicly available at our website [42].

## Authors' contributions

TJ contributed in the data collection, sequence alignments and generation and analysis of the results. Both TJ and DX contributed in the formulation, design and writing of the study. Both authors read and approved the final manuscript.
